# Proteomic Profiling of Ectosomes Derived from Paired Urothelial Bladder Cancer and Normal Cells Reveals the Presence of Biologically-Relevant Molecules

**DOI:** 10.3390/ijms22136816

**Published:** 2021-06-24

**Authors:** Magdalena Surman, Sylwia Kędracka-Krok, Urszula Jankowska, Anna Drożdż, Ewa Stępień, Małgorzata Przybyło

**Affiliations:** 1 Department of Glycoconjugate Biochemistry, Institute of Zoology and Biomedical Research, Faculty of Biology, Jagiellonian University in Kraków, 30-387 Kraków, Poland; magdalena.surman@uj.edu.pl; 2 Department of Physical Biochemistry, Faculty of Biochemistry, Biophysics and Biotechnology, Jagiellonian University in Kraków, 30-387 Kraków, Poland; sylwia.kedracka-krok@uj.edu.pl; 3 Proteomics and Mass Spectrometry Core Facility, Malopolska Centre of Biotechnology, Jagiellonian University in Kraków, 30-387 Kraków, Poland; urszula.jankowska@uj.edu.pl; 4 Department of Medical Physics, M. Smoluchowski Institute of Physics, Faculty of Physics, Astronomy and Applied Computer Science, Jagiellonian University in Kraków, 30-348 Kraków, Poland; anna.drozdz@uj.edu.pl (A.D.); e.stepien@uj.edu.pl (E.S.)

**Keywords:** biomarkers, bladder cancer, ectosomes, extracellular vesicles, nanoLC-MS/MS, mass spectrometry, proteomics

## Abstract

Protein content of extracellular vesicles (EVs) can modulate different processes during carcinogenesis. Novel proteomic strategies have been applied several times to profile proteins present in exosomes released by urothelial bladder cancer (UBC) cells. However, similar studies have not been conducted so far on another population of EVs, i.e., ectosomes. In the present study we used a shotgun nanoLC–MS/MS proteomic approach to investigate the protein content of ectosomes released in vitro by T-24 UBC cells and HCV-29 normal ureter epithelial cells. In addition, cancer-promoting effects exerted by UBC-derived ectosomes on non-invasive cells in terms of cell proliferation and migratory properties were assessed. In total, 1158 proteins were identified in T-24-derived ectosomes, while HCV-29-derived ectosomes contained a lower number of 259 identified proteins. Qualitative analysis revealed 938 proteins present uniquely in T-24-derived ectosomes, suggesting their potential applications in bladder cancer management as diagnostic and prognostic biomarkers. In addition, T-24-derived ectosomes increased proliferation and motility of recipient cells, likely due to the ectosomal transfer of the identified cancer-promoting molecules. The present study provided a focused identification of biologically relevant proteins in UBC-derived ectosomes, confirming their role in UBC development and progression, and their applicability for further biomarker-oriented studies in preclinical or clinical settings.

## 1. Introduction

Urothelial bladder carcinoma (UBC) is one of the most frequent urological malignancies worldwide, with morbidity and mortality rates of 424,000 new cases and 200,000 deaths per year [1]. The main diagnostic approaches in UBC include cystoscopy and urinary cytology. However, due to their limited sensitivity and specificity, both methods are considered fully effective only in more advanced stages of UBC [2]. Despite ongoing technological advancements (such as development of white and blue light cystoscopy [3]), alternative, non-invasive diagnostic and surveillance methods for UBC are still needed. Current non-invasive tests for UBC target a variety of urinary proteins or nucleic acids [2,3]. Some are commercialized, but their sensitivities and specificities are insufficient to replace cystoscopy in standard diagnostic protocols.

Recently, a lot of attention in the biomarker field has been given to extracellular vesicles (EVs), namely exosomes (having endosomal origin) and ectosomes (membrane-derived, also called microvesicles). These small, membrane-enclosed structures are released by almost all cell types and participate in intercellular transfer of bioactive molecules such as proteins, lipids and nucleic acids. Once EVs are released to the extracellular space, they can be isolated from a variety of body fluids, mainly blood, urine and cerebrospinal fluid, or from conditioned media in cell culture experiments. Such bioavailability and the dependence of vesicular cargo on the cell molecular content together make EVs a promising target in the search for novel biomarkers in many urological cancers, including UBC [2,4,5].

Past research has shown that UBC-derived exosomes can directly modulate particular processes during carcinogenesis, such as cancer cell migration [6], epithelial–mesenchymal transition (EMT) [7], angiogenesis [6] and inhibition of apoptosis [8]. In addition to the functional effects, a higher concentration of exosomes was observed in urine of UBC patients compared to that of healthy individuals [9]. In addition, up-to-date proteomic strategies were applied several times to profile the very dynamic range of proteins present in UBC-derived exosomes [6,10,11,12,13,14,15,16,17,18]. Multiple, potential protein markers of UBC were identified, although their clinical relevance is yet to be determined.

So far, similar studies have not been conducted on the latter population of EVs. Nevertheless, ectosomes can also contain a specific protein profile enriched in cancer biomarkers; this was demonstrated in our previous studies for melanoma [19,20] as well as other studies on breast [21], head and neck [22], and ovarian cancer [23]. In the present study we used a liquid chromatography coupled with tandem mass spectrometry-based (nanoLC–MS/MS) proteomic approach to investigate the protein content of ectosomes released in vitro by T-24 UBC and HCV-29 normal ureter epithelial cells. In addition, cancer-promoting effects exerted by UBC-derived ectosomes on non-invasive cells in terms of cell proliferation and migratory properties were assessed.

## 2. Results

### 2.1. Asessment of Purity of Ectosome Samples

**Figure 1:**
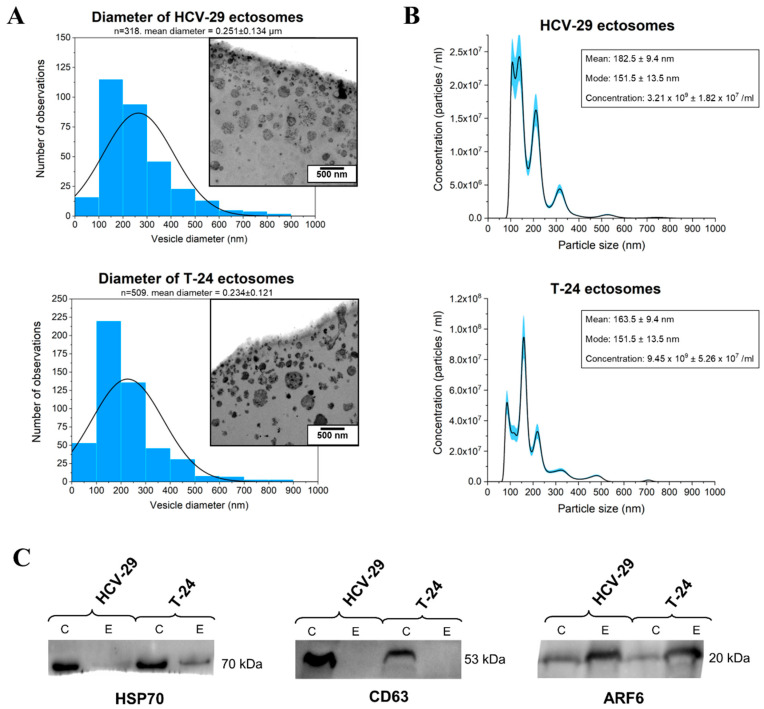
Characterization of ectosome samples isolated from conditioned media of T-24 urothelial bladder carcinoma and HCV-29 normal ureter epithelial cells. (**A**) Morphological characterization of HCV-29- and T-24-derived ectosomes by transmission electron microscopy (TEM). Size distributions are presented on histograms. Mean diameter ± standard deviation was calculated for all observed vesicles (*n*) from a given sample. (**B**) Nanoparticle tracking analysis (NTA) of HCV-29- and T-24-derived ectosomes. Results from five independent measurements for each cell line are presented on graphs. The shaded area depicts standard deviation. (**C**) Representative Western blot of extracellular vesicle markers in whole-cell protein extracts (lines C) and ectosome samples (lines E). Fifty μg of proteins separated by 10% SDS-PAGE and transferred into PVDF membrane were probed with anti-HSP70 (1:2000), anti-CD63 (1:2000) and anti-ARF6 (1:500) as primary antibodies and anti-mouse IgG-HRP (1:400) as a secondary antibody.

The purity of T-24- and HCV-29-derived ectosome samples obtained after 18,000× *g* centrifugation was assessed using transmission electron microscopy (TEM) and nanoparticle tracking analysis (NTA). In TEM images (Figure 1A) no contamination with cells or cellular organelles was observed. Isolated populations of ectosomes were found to be rather heterogeneous in size and most of the vesicles were in the predefined diameter range for ectosomes, i.e., 100–1000 nm. Analogous samples were also analyzed by NTA (Figure 1B). The obtained results again confirmed that a majority of isolated EVs were over 100 nm in diameter, demonstrating that contamination of the isolated sample with exosomes was negligible. The most numerous subpopulation of ectosomes in each EV sample consisted of ectosomes with a diameter in the range of 100–300 nm as shown by both TEM and NTA analysis.

Additionally, the purity of ectosome sample isolation was verified by Western blot (WB). WB analysis revealed the absence or depletion of CD63 and HSP70, which are classical exosomal protein markers, in isolated EV samples (Figure 1C). At the same time, it was shown that all EV samples were enriched in ARF6, the protein which is thought to regulate ectosome, but not exosome, biogenesis. Therefore, we considered the isolated EV samples to be highly enriched in ectosomes and such samples were used for further experiments.

### 2.2. Proteins Identified in T-24- and HCV-29-Derived Ectosomes—Qualitative and Quantitative Analysis

**Figure 2:**
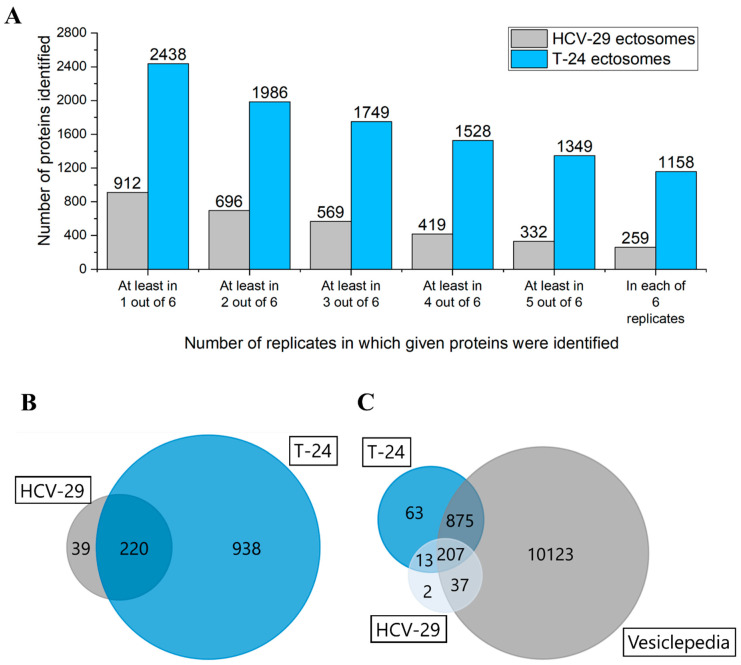
(**A**) Number of proteins identified in particular numbers of replicates of ectosome samples (the total number of six replicates included two biological replicates with three technical replicates each). (**B**) Venn diagram illustrating the number of proteins identified in two biological replicates of ectosomes derived from T-24 urothelial bladder carcinoma and HCV-29 normal ureter epithelial cells by at least two peptides. (**C**) Venn diagram illustrating protein overlap between isolated ectosomes and Vesiclepedia database as a reference.

Gel-free shotgun nanoLC–MS/MS proteomic approach was used to profile protein content of T-24- and HCV-29-derived ectosomes. In total, 1158 proteins were identified in all biological and technical replicates of T-24-derived ectosomes, while HCV-29-derived ectosomes contained a significantly lower number of 259 identified proteins (Figure 2A). Complete lists of proteins identified in all particular replicates can be found in Supplementary Data S1. A common set of 220 proteins was stated in ectosomes released by cells of both cell lines tested. The majority of proteins identified by the present study (93.4% for T-24-derived ectosomes, 94.2% for HCV-29-derived ectosomes) were also found by other vesicle-related studies, as shown by comparison to the Vesiclepedia database (Figure 2B). It strongly supports their vesicular origin and proves that they are not a part of co-isolated cell debris or remnants of conditioned media.

**Figure 3:**
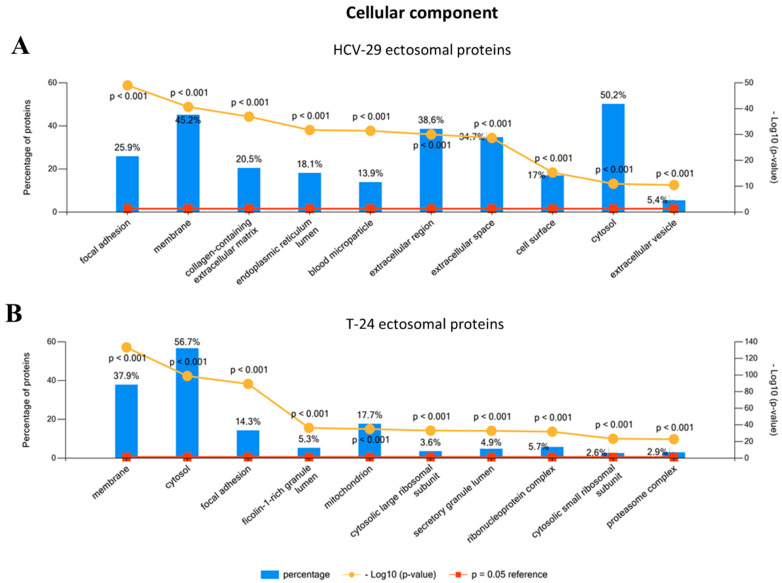
Gene ontology (GO) analysis of proteins identified in ectosomes derived from HCV-29 normal ureter epithelial cells (**A**) and T-24 urothelial bladder carcinoma cells (**B**) performed with the use of FunRich 2.0 software with UniProt (release 2020_12) database as a reference. For GO term “Cellular compartment” ten categories with the highest statistical significance of protein enrichment within the specific category (*p* < 0.001) were presented on graphs. Complete results of GO analysis are provided in Supplementary Data S2.

Proteins identified in ectosome samples were then grouped according to the Gene Ontology (GO) aspects (cellular compartment, molecular function, biological process) with the use of FunRich 2.0 software with UniProt (release 2020_12) database as a reference (Supplementary Data S2). For ectosomes derived from both cell lines, the most numerous groups of ectosomal proteins were those of cytosolic (up to 56.7% of identified proteins) or membrane origin (up to 45.2%) (Figure 3). These results are in line with the mechanism of ectosome biogenesis in which fragments of cytoplasm are surrounded by adjacent regions of the plasma membrane.

**Figure 4:**
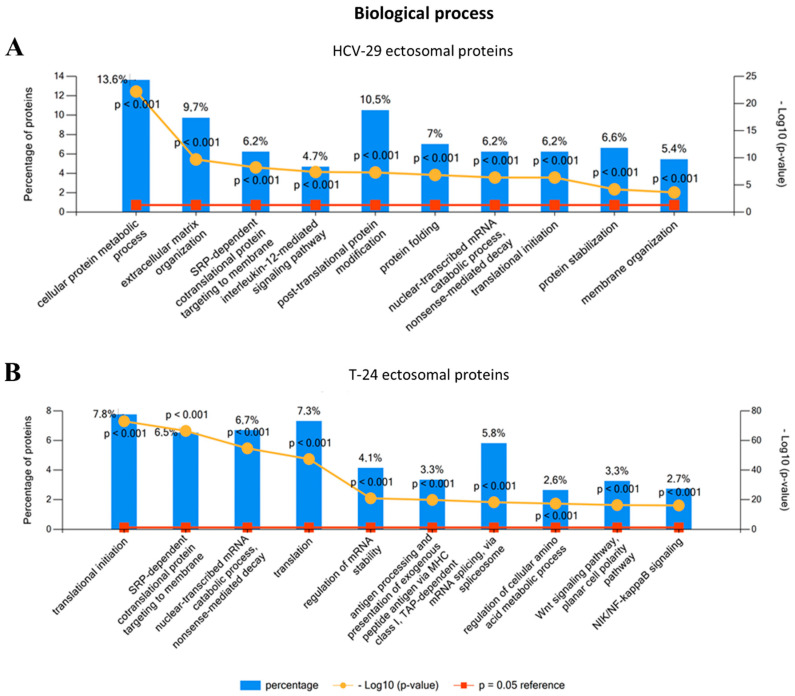
Gene ontology (GO) analysis of proteins identified in ectosomes derived from HCV-29 normal ureter epithelial cells (**A**) and T-24 urothelial bladder carcinoma cells (**B**) performed with the use of FunRich 2.0 software with UniProt (release 2020_12) database as a reference. For GO term “Biological process” ten categories with the highest statistical significance of protein enrichment within the specific category (*p* < 0.001) were presented on graphs. Complete results of GO analysis are provided in Supplementary Data S2.

As far as biological processes are concerned, HCV-29-derived ectosomes mostly contained proteins involved in cellular metabolism, post-translational protein modification as well as protein folding, stabilization, and targeting (Figure 4A). On the other hand, the most numerous GO terms for T-24-derived ectosomal proteins refer to translational initiation and related processes such as mRNA splicing, stabilization, and metabolism (Figure 4B). This may suggest the regulatory role of T-24-derived ectosomes during translation and subsequent protein biosynthesis in recipient cells. Further, enrichment within categories related to cancer-promoting signaling pathways, i.e., Wnt and NIK/NF-kappaB, was reported for T-24-derived ectosomes, reflecting their cancerous origin. Finally, multiple proteins involved in antigen presentation via MHC class I molecules were present in T-24-derived ectosomes. It may be one of the mechanisms through which cancer cells escape from the immune system surveillance.

**Figure 5:**
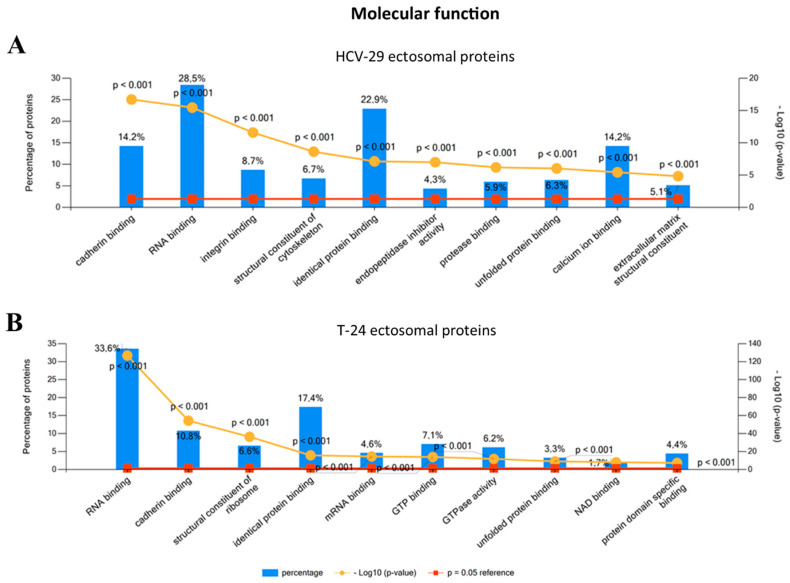
Gene ontology (GO) analysis of proteins identified in ectosomes derived from HCV-29 normal ureter epithelial cells (**A**) and T-24 urothelial bladder carcinoma cells (**B**) performed with the use of FunRich 2.0 software with UniProt (release 2020_12) database as a reference. For GO term “Molecular function” ten categories with the highest statistical significance of protein enrichment within the specific category (*p* < 0.001) were presented on graphs. Complete results of GO analysis are provided in Supplementary Data S2.

When classified by their precise molecular functions, a significant number of identified proteins from HCV-29- and T-24-derived ectosomes had nucleic acid (mainly RNA) and protein binding activity (Figure 5). In addition, T-24-derived ectosomes were enriched in nucleotide binding proteins. This suggests that ectosomes may play a significant role in the coordination of different signaling pathways in recipient cells, which often involve nucleotides as signaling molecules.

Next, we performed normalized spectral counting-based quantitative analysis of proteins unique for HCV-29- and T-24-derived ectosome samples for their better characterization, using the methodology described in [24]. Spectral counts of each protein were used to estimate protein abundance in a distributive manner, i.e., peptide spectral counts were calculated for each protein based on unique peptides and a weighted distribution of any peptide shared with homologous proteins. Normalization was done for proteins’ length and whole protein content in the sample (Supplementary Data S3).

**Figure 6:**
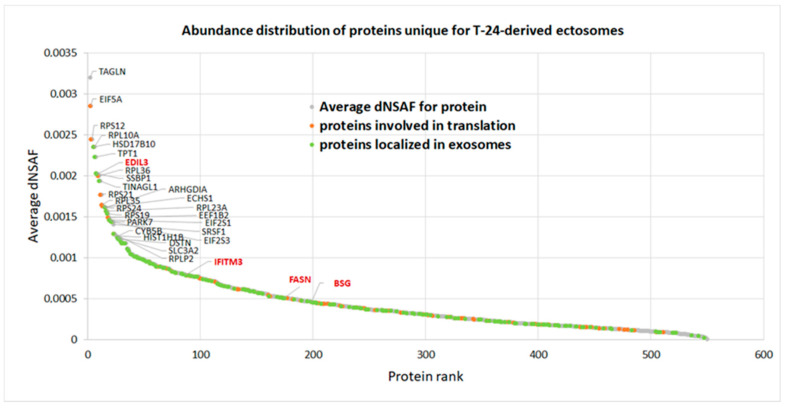
Normalized spectral counting-based abundance of 549 proteins identified uniquely in T24-derived ectosomes (not identified in any replicate of HCV-29-derived ectosomes). The most abundant proteins are labeled. Detailed results can be found in Supplementary Data S3. (dNSAF—distributed normalized spectrum abundance factor).

Figure 6 depicts the relative abundance of proteins uniquely identified in T-24-derived ectosomes (i.e., not identified in any replicate of HCV-29-derived ectosomes). In total, 180 out of 549 proteins were assigned by GO to exosomal localization, including EGF-like repeat and discoidin I-like domain-containing protein 3 (EDIL3), interferon-induced transmembrane protein 3 (IFITM3), fatty acid synthase (FASN) or basigin (BSG). Although in the present study ectosomes (not exosomes) were isolated, a variety of proteins can be found in more than one EVs population, thus assignment of the aforementioned proteins to exosomal localization by GO only demonstrates their vesicular origin. Furthermore, many (116) of the proteins unique for T-24-derived ectosomes were assigned by GO to the protein translation process. Ribosomal proteins and translation factors also dominated among the most abundant proteins in T-24-derived ectosomes.

**Figure 7:**
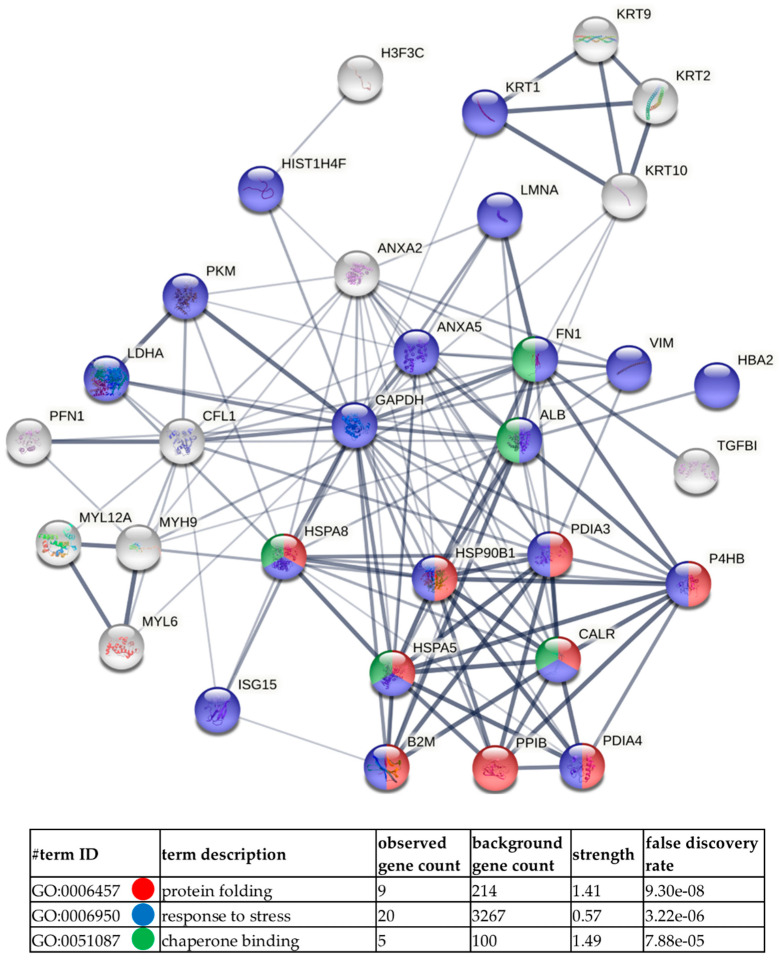
Diagram of functional protein association networks prepared with the use of STRING v. 11.0 software for 10% of the most abundant proteins identified in HCV-29-derived ectosomes (i.e., 33 proteins). The selected strongly represented pathways are presented on interactome. Complete results, including proteins that were not identified in all biological/technical replicates, are provided in Supplementary Data S3.

**Figure 8:**
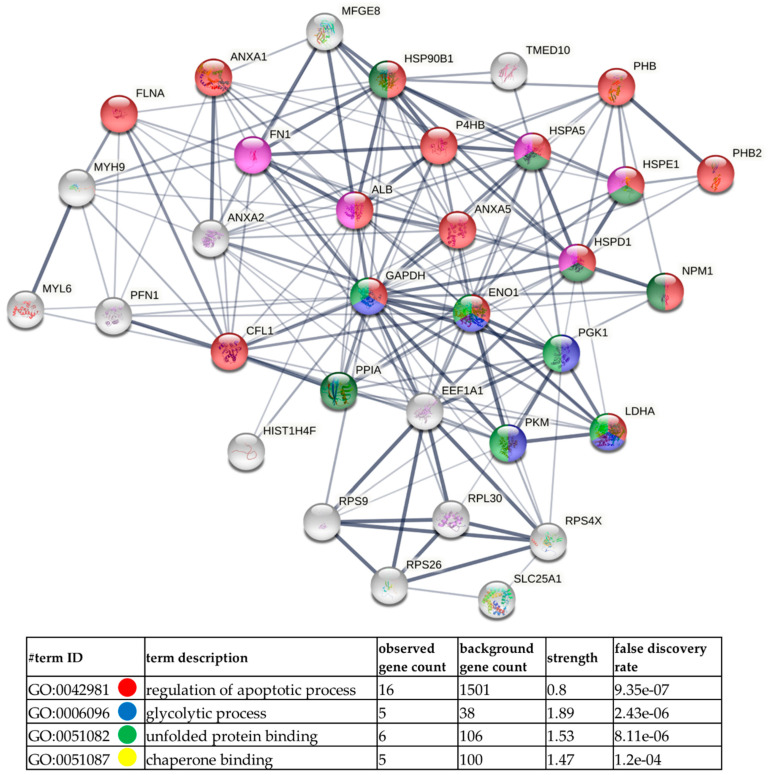
Diagram of functional protein association networks prepared with the use of STRING v. 11.0 software for 33 (~2.5%) of the most abundant proteins identified in T-24-derived ectosomes. The selected strongly represented pathways are presented on interactome. Complete results, including much more complex networks with 10% of most abundant proteins (135 proteins), are provided in Supplementary Data S3.

Next, GO analysis and diagrams of functional protein association networks were prepared with the use of STRING v. 11.0 software. For HCV-29-derived ectosomes 10% of the most abundant proteins based on dNSAFs that were identified in five or six replicates were taken into consideration (i.e., 33 proteins). The most strongly represented pathways included protein folding and chaperon binding which relate to another enriched GO category, namely, response to stress (Figure 7). Analogously, 33 of most abundant proteins from T-24-derived ectosomes were analyzed (Figure 8), revealing enrichment in proteins related to unfolded protein binding and chaperon binding. T-24-derived ectosomes were also highly enriched in proteins involved in apoptosis regulation and glycolytic process, which may result from the Warburg effect often observed in cancer cells. Finally, when 10% of the most abundant proteins (135 of 1349) for T-24-derived ectosomes were analyzed (Supplementary Data S3), the most enriched pathways included ribosomal constituents as well as translational and co-translational processes. This suggests the involvement of T-24-derived ectosomal proteins in widely understood protein biosynthesis.

Finally, the results of bioinformatic analysis of 938 proteins identified uniquely in T-24-derived ectosomes reflect their cancerous origin. Proteins assigned to the chosen cancer-related GO categories with the use of UniProt (release 2020_12) database are listed in Table 1. They include proteins involved in cancer cell proliferation, adhesion and migration, angiogenesis as well as immune and drug response.

### 2.3. Functional Effect of T-24- and HCV-29-Derived Ectosomes on Recipient Cells

Due to the fact that numerous cancer-promoting proteins were identified in T-24-derived ectosomes by nanoLC-MS/MS, functional tests were performed to assess whether protein cargo of UBC-derived ectosomes indeed modulate the function of recipient cells. Changes in cell viability and motility were evaluated after 18 h of incubation of normal and UBC cells with T-24-derived ectosomes as well as HCV-29-derived ectosomes as a control.

**Figure 9:**
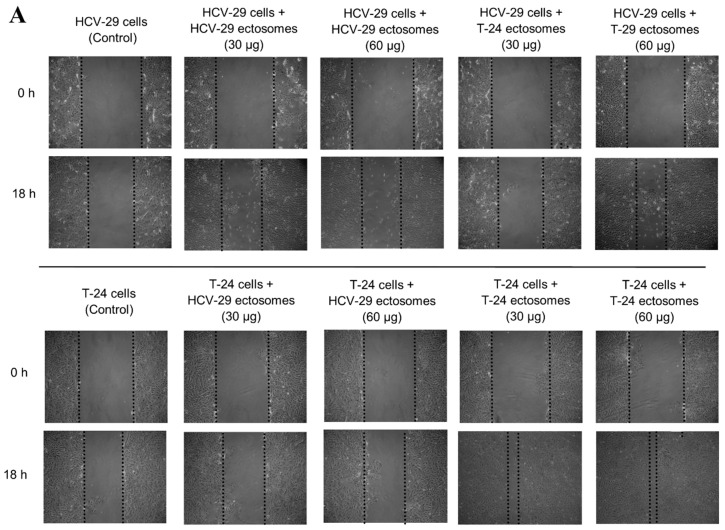

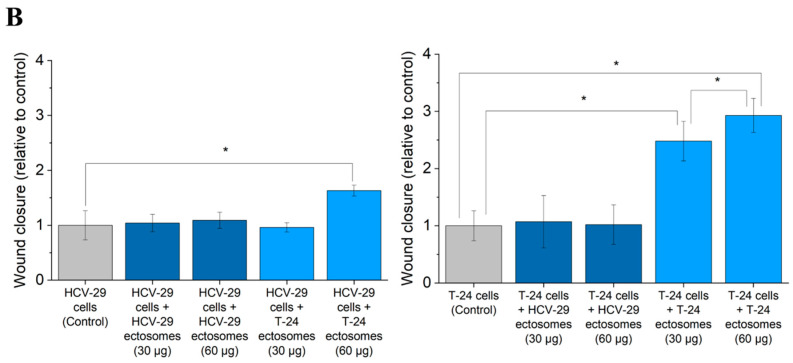
Effect of incubation of T-24 urothelial bladder carcinoma and HCV-29 normal ureter epithelial cells with ectosomes released by these cell lines. Wound healing assay was performed after 18 h of incubation with ectosomes. (**A**) Representative images were taken at 0 h and at 18 h. (**B**) Graphs presenting the relative rate of wound closure calculated from three repetitions. “*” denotes statistically significant differences (Tukey’s post-hoc test, *p*-value < 0.05).

In wound healing assay, the extent of wound closure in control conditions (cells without ectosomes) was 19% for HCV-29 cells and 32% for T-24 cells. The addition of T-24-derived ectosomes increased the motility of recipient T-24 and HCV-29 cells (Figure 9). The stronger and dose-dependent response was observed in the case of T-24 cells (2.47-fold and 2.94-fold increase when adding 30 µg and 60 µg of ectosomal proteins, respectively), whereas HCV-29 cells displayed a weaker increase in motility (1.7-fold only) when treated with a higher dose of T-24-derived ectosomes. On the contrary, ectosomes derived from normal HCV-29 cells did not induce any significant response in either of the cell lines.

To sum up, ectosomes derived from cancerous T-24 cells displayed a higher ability to boost viability and motility of both recipient cell lines in comparison to ectosomes derived from non-transformed HCV-29 cells. Therefore, bladder cancer-derived ectosomes should be considered as one of the factors promoting the progression of the existing tumors. To some degree they may also affect the function of normal epithelial cells within the tumor microenvironment.

**Figure 10:**
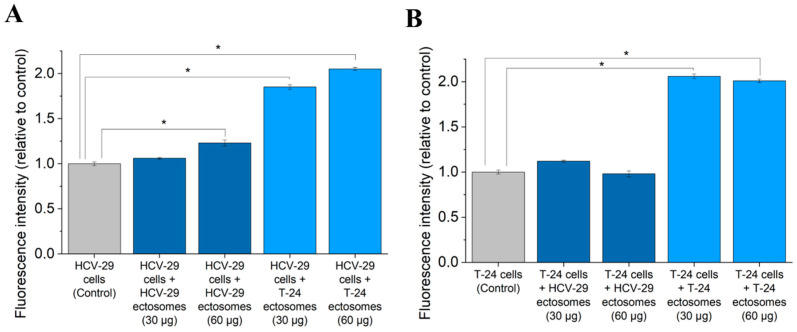
Effect of incubation of T-24 urothelial bladder carcinoma (**A**) and HCV-29 normal ureter epithelial cells (**B**) with ectosomes released by these cell lines. Alamar Blue cell viability assay was carried out after 18 h of incubation with ectosomes. All experiments were conducted in triplicate. “*” denotes statistically significant differences (Tukey’s post-hoc test, *p*-value < 0.05).

Additionally, ectosome-induced changes in viability of recipient T-24 and HCV-29 cells were assessed using Alamar Blue cell viability assay (Figure 10). An approximately two-fold increase in fluorescence intensity was observed after the addition of T-24-derived ectosomes to either T-24 or HCV-29 cells, but the effect was not dose-dependent. Further, a higher dose of HCV-29-derived ectosomes increased the viability of HCV-29 cells but did not affect T-24 cells.

## 3. Discussion

### 3.1. Proteins Cargo of UBC-Ectosomes Is Enriched in Proteins Involved in Cancer Progression

In the present study nanoLC–MS/MS proteomic approach was applied to analyze the protein content of ectosomes released in vitro by T-24 UBC and HCV-29 normal ureter epithelial cells. Ectosomes were isolated from conditioned media collected after 24 h of culture in serum-free media to avoid the contamination with sera-derived proteins (mostly albumins) and sera-derived EVs. Then we applied our previously established protocol for sequential centrifugation [19,20] with three steps at low g-force, allowing to remove remaining cells, cell debris and larger particles (such as apoptotic bodies), and with the final step at 18,000× *g* which allowed to pellet ectosomes. Indeed, g-force of 18,000× *g* is sufficient for isolation of ectosomes, but insufficient for pelleting the majority of exosomes (over 100,000× *g* is required). However, due to the size overlap between the two EV subpopulations (particularly vesicles with a diameter of approx. 100 nm), complete separation thereof is difficult, and the samples should rather be described in terms of relative exosome/ectosomes enrichment. In the present study we used TEM and NTA which showed that the majority of isolated EVs were within the predefined size range for ectosomes (>100 nm in diameter). They were also depleted of exosomal markers (CD63 and Hsp70), and enriched in ARF6, a protein marker confirming the plasma membrane origin of ectosomes. Based on the above evidence, we considered the isolated EV population to be highly enriched in ectosomes.

In all EV-oriented studies development of proper and efficient protocols for EV isolation forms the basis for further investigation regarding either EV molecular content or their functional role in different physiological and pathological processes. Numerous studies have shown that EV-mediated transfer of bioactive molecules, including proteins, affects various stages of cancer progression by altering the communication between tumor cells and tumor microenvironment. Delivery of EV protein cargo to recipient cells may promote their neoplastic transformation, proliferation, migration, invasion, and subsequent angiogenesis. In the case of UBC, it was demonstrated that exposure of normal urothelial cells to UBC-derived exosomes induced EMT, after which the cells gained migratory and invasive properties. [7]. In addition, treatment of 5637 and T-24 UBC cells with T-24-derived exosomes stimulated their proliferation in a dose- and time-dependent manner, most likely due to activation of protein kinase B (Akt) and extracellular signal–regulated kinase (ERK) signaling pathways [7]. In addition, quantitative iTRAQ proteomic analysis of T-24-derived exosomes was performed by Jeppesen et al. [18]. They identified 1587 proteins, several of which, such as vimentin, hepatoma-derived growth factor, casein kinase II α and annexin A2, are directly linked to EMT, and were also identified in T-24-derived ectosomes in the present study. Interestingly, the studies by Jeppesen et al. [18] also showed the increased abundance of the aforementioned proteins in exosomes derived from isogenic cells lines obtained either from lung or liver metastasis in mice previously inoculated with T-24 cells. This suggests that EV proteins are directly involved in the metastatic process, particularly in the location of secondary tumors.

In correspondence to the above-mentioned studies on exosomes, we observed similar cancer-promoting effects exerted by ectosomes in wound healing and Alamar Blue assays. Ectosomes derived from cancerous T-24 cells displayed a higher ability to boost viability (proliferation) and motility of recipient cell lines in comparison to ectosomes derived from non-transformed HCV-29 cells. Therefore, like exosomes, UBC-derived ectosomes and their protein cargo should be considered as one of the factors promoting the progression of the existing UBC tumors.

T-24-derived ectosomal proteins that might be potentially responsible for the observed effects were selected by GO ontology analysis and included mostly signaling and adhesion (or adhesion-related) molecules (Table 1). Among others, β-catenin was identified, a crucial factor of Wnt/β-catenin signaling pathway. Chen et al. [25] recently demonstrated that activation of Wnt/β-catenin pathway promotes in vitro migration and invasion of EJ and T-24 UBC cells. Moreover, conditioned medium from UBC cells stimulated tubule formation by human umbilical vein endothelial cells (HUVECs), suggesting that UBC secretome (including ectosomes and other EVs) may exert a functional effect during angiogenesis [25].

Moreover, collagens are another component of the tumor microenvironment closely associated with EVs. Exosomes were shown to induce the differentiation of cancer-associated fibroblasts (CAFs) in the collagen-rich extracellular matrix (ECM) [26] and to secrete collagen-regulatory factors such as matrix metalloproteinase-14 (MMP-14) [27], whereas collagen type I enhanced exosome secretion [28]. Different collagens (type I, IV, V, VI, XII and XVIII) were identified in T-24-derived ectosomes in the present study, proving that EVs can also be carriers of collagen molecules. Particularly in UBC, type VI collagen has already been implicated in the promotion of EMT [29], while collagens IV and XIII were associated with increased primary tumor budding and invasiveness [30].

To some degree, UBC-derived ectosomes may also affect the function of normal epithelial cells within the tumor microenvironment, as shown in the present study for HCV-29 cells treated with T-24-derived ectosomes. Similarly, Goulet et al. reported that exosomes derived from RT4, T-24 and SW1710 UBC cells can promote transformation of normal fibroblasts into CAFs, displaying increased proliferation and migration rates as well as elevated expression of chosen CAF markers [31].

Furthermore, tumor-derived EVs are known to alter the immune response towards cancer cells either by activation or, more often, suppression of the immune system. One of the aspects is the loss of MHC molecules via EVs which may impede the presentation of tumor-associated antigens on the cell surface and favor escape from immune surveillance. Indeed, several proteins involved in antigen presentation via MHC class I molecules were identified in T-24-derived ectosomes in the present study. Secondly, EV protein cargo may inhibit the action of the effector cells such as NK cells towards cancer cells. Lee et al. identified mucin 1 (MUC1) and carcinoembryonic antigen (CEA) to be upregulated in urinary exosomes from UBC patients; both these proteins are known to contribute to NK cell evasion by cancer cells [17]. In addition, sphingosine 1-phosphate receptor (S1P1R) was identified in T-24-derived ectosomes. S1P1R promotes regulatory T cells (Treg) accumulation within tumors, which act to suppress immune response [32]. Finally, inflammatory conditions contribute to malignant progression through the recruitment and activation of inflammatory cells. Proinflammatory factors such as interleukin 18, apolipoprotein B (ApoB) and transaldolase 1 (TALDO1) were present in T-24-derived ectosomes and previously identified by other studies in UBC exosomes [14,33].

### 3.2. UBC-Derived Ectosomes as Potential Carriers of Clinically-Relevant Proteins

Bioavailability of EVs in different body fluids makes them a promising, non-invasive source of biomarkers in numerous diseases, including cancer. It has been shown that the changes in the number and/or protein content of EVs released by cancer cells may reflect their pathological status. Cancer cells are known to secrete more EVs than normal cells. For instance, Liang et al. demonstrated a significantly higher concentration of CD63-positive EVs (presumably exosomes) in urine from patients with UBC compared to urine of healthy controls [9]. Similarly, in the present study NTA analysis revealed a higher concentration of T-24-derived ectosomes in comparison to those derived from HCV-29 cells after equal volumes of conditioned media were taken for ectosome isolation.

Recently, there has been a growing number of studies using proteomic profiling of EVs in search for molecular markers of UBC. The advantage of using urinary EVs over whole urine samples was demonstrated by Lee et al., who identified 1.5 times more proteins in urinary EVs than in the whole urine samples from UBC patients [17]. These results were explained by significantly lower levels of albumin in EV samples that did not mask the identification of low-abundant proteins by LC-MS/MS. In the present study almost 4.5 times more proteins were identified in UBC-derived ectosomes in comparison to ectosomes derived from normal cells. This is in line with another finding made by Lee et al. that EVs from UBC patients displayed higher protein yield than those from healthy individuals [17].

So far exosomes were the only EV population studied in the search for novel UBC protein biomarkers. The present study provides the very first proteomic insight into the diagnostic potential of ectosomes in this type of cancer. Around 5% of unique proteins identified in all samples of UBC T-24-derived ectosomes showed strong identification with the number of unique peptides >15 and peptide-spectrum match (PMS) >15 (Supplementary Data S3). Among those proteins, FASN, IFITM3 and EDIL3 were some of the most abundant. It has already been shown that downregulation of FASN expression or inhibition of FASN molecule prevents AKT kinase phosphorylation. Preventing AKT phosphorylation through FASN exerted anti-proliferative and anti-migratory effects on UBC cells [34,35,36], suggesting that ectosomal FAS may also be a potential therapeutic target in UBC.

On the other hand, EDIL3 (EGFR-activating, proangiogenic integrin ligand) was previously identified in exosomes from invasive bladder cancer cell lines and from urine of patients with high-grade UBC [6]. EDIL3 levels were higher in patients’ urinary exosomes than in exosomes from healthy individuals, suggesting their diagnostic potential. Moreover, *EDIL3* knockout and gene silencing in functional tests proved the direct involvement of exosomal EDIL3 in migration of UBC and endothelial cells as well as in angiogenesis. In the present study, EDIL3 was identified in T-24-derived ectosomes and might be one of the factors contributing to their pro-migratory and proangiogenic potential. Other proteins identified in T-24-derived ectosomes that have already been described as differentially expressed in UBC exosomes include two more proteins related to epidermal growth factor receptor (EGFR) signaling pathway (i.e., Eps15 Homology (EH)-domain-containing protein 4 (EHD4) and epidermal growth factor receptor kinase substrate 8-like protein 2 (EPS8L2)), both of which are known to be downregulated in UBC exosomes [12].

The LC-MS/MS quantitative approach was also applied by Chen et al. [13] who identified 24 proteins differentially expressed in urinary exosomes from UBC patients in comparison to control samples. One of these proteins, i.e., tumor-associated calcium-signal transducer 2 (TACSTD2), was further evaluated with commercial ELISA kits that confirmed TACSTD2 diagnostic potential. Interestingly, TACSTD2 was identified in T-24-derived ectosomes by the present study, but was absent in ectosomes derived from normal HCV-29 cells, proving that this population of EVs may also present a diagnostic value in UBC.

T-24-derived ectosomes also contained extracellular matrix metalloproteinase inducer (EMMPRIN, also called basigin or CD147), and ecto-5′-nucleotidase (NT5E or CD73), elevated levels of which have already been observed in urinary exosomes derived from UBC patients [16]. Interestingly, CD147 was uniquely identified only in T-24-derived ectosomes; NT5E, however, was present in all samples. Both CD147 and NT5E can promote invasion and metastasis of cancer cells by signaling (for NT5E) with ECM components such as fibronectin and laminin, or by their degradation (CD147). In addition, free adenosine generated by NT5E inhibits cellular immune responses and might enable tumor cells to evade immune surveillance. NT5E is therefore being considered as a potential drug target in cancer immunotherapy [16].

Furthermore, periostin, which functions as a ligand for alpha V/beta 3 and alpha V/beta 5 integrins to support adhesion and migration of epithelial cells and is associated with poor clinical outcome in bladder cancer, was also identified in T-24-derived ectosomes. Previously, a proteomic study by Silvers et al. found periostin to be abundant in exosomes derived from highly invasive UBC TCC-SUP cells but absent in exosomes derived from normal urothelial cell line SV-HUC [15]. Further, treatment of less invasive UBC cells with periostin-rich EVs promoted cell aggressiveness by activation of ERK oncogenic signals. The potential of periostin as a UBC biomarker was confirmed by significantly higher levels of periostin measured in UBC patients’ urinary exosomes compared to those from healthy individuals. Apart from periostin, Silvers et al. [15] found five more potential biomarkers in UBC exosomes, four of which were also identified in T-24-derived ectosomes in the present study, i.e., beta-hexosaminidase subunit beta (HEXB), staphylococcal nuclease domain-containing protein 1 (SND1), transaldolase 1 (TALDO1), and EH domain containing 4 (EHD4).

T-24-derived ectosomes also contained histone cluster 1 H2B family member K (H2B1K). In study by Lin et al., detection of H2B1K in patients’ urinary exosomes correlated with a higher risk of recurrence and progression stage of UBC determined by tumor tissue immunohistochemical staining [11]. Lastly, Lee et al. presented the list of 20 up-regulated proteins (based on the raw spectral counts method) in urinary EVs derived from bladder cancer patients [17]. Some of these proteins, i.e., EHD4, ES8L2, ezrin, 60 kDa heat shock protein (Hsp60), myosin-9, subunit beta of beta-hexosaminidase, and 14-3-3 protein epsilon isoform, were also identified in T-24-derived ectosomes in the present study.

Prognosis for UBC patients remains poor despite the constant improvement of diagnostic methods and development of new therapies. Progressive research on EVs in UBC and other urological cancers holds promise for a deeper understanding of disease biogenesis and pathogenesis, and might provide novel therapeutic and diagnostic targets. The present study provided a focused identification of biologically relevant proteins in UBC-derived ectosomes, proving applicability of this particular EV population for further studies in preclinical or clinical settings.

## 4. Materials and Methods

### 4.1. Materials

RPMI 1640 GlutaMAX™-I medium, fetal bovine serum (FBS), MicroBCA Protein Assay kit, Alamar Blue cell viability reagent, Acclaim PepMap trap (100 C18, 75 μm × 20 mm, 3 μm particle, 100 Å pore size) and analytical (Acclaim PepMap RSLC C18, 75 µm × 500 mm, 2 µm particle, 100 Å pore size) columns were all purchased from Thermo Fisher Scientific (Waltham, MA, USA). Anti-CD63 mouse monoclonal primary antibody (clone RFAC4, cat. CBL553), Lumi-LightPLUS Western Blotting Kit (including anti-mouse IgG-HRP secondary antibody), Trypsin-EDTA solution, penicillin/streptomycin solution, SpeedBeads™ GE45152105050250 and GE65152105050250, and HEPES buffer were obtained from Sigma-Aldrich (St. Louis, MO, USA). Mouse monoclonal primary antibodies for ARF6 (clone 3A-1, cat. sc-7971) and HSP70 (clone C92F3A-5, cat. sc-66048) were purchased from Santa Cruz Biotechnology (Dallas, TX, USA). Trypsin/Lys-C Mix was the product of Promega (Madison, WI, USA). Other chemicals were of analytical grade, commercially available.

### 4.2. Cell Lines and Cell Culture Conditions

Human bladder cancer cell lines (T24) [37] and human non-malignant ureter epithelium cell line (HCV29) [38] were kindly donated by Professor Danuta Duś, the Institute of Immunology and Experimental Therapy, Polish Academy of Sciences, Wrocław, Poland. The cells were maintained in RPMI 1640 medium with GlutaMAX-I, supplemented with 10% FBS, penicillin (100 unit/mL) and streptomycin (100 μg/mL). The cells were grown in monolayers in a 5% CO_2_ atmosphere at 37 °C in a humidified incubator and passaged after reaching approximately 80% confluence.

### 4.3. Isolation of Ectosomes and Characterization of Isolated Samples

Sub-confluent cells were cultured for 24 h in serum-free medium. Conditioned media were collected and subjected to sequential centrifugation steps. After centrifugations at 400× *g* (5 min, 4 °C), 4000× *g* (20 min, 4 °C) and 7000× *g* (20 min, 4 °C), the remaining cells and cellular debris were pelleted and discarded, whereas supernatants were collected for ectosome isolation. After final centrifugation at 18,000× *g* (20 min, 4 °C), ectosomes were pelleted and resuspended in ice-cold PBS.

The purity of the obtained samples was verified by transmission electron microscopy (TEM) as previously described [19] as well as by nanoparticle tracking analysis (NTA). NTA measurements were performed on NanoSight LM 10 (Malvern Panalytical) equipped with a 405 nm laser. For NTA analysis, 10 µL of each ectosome sample was diluted to 2 mL with filtered PBS. The measurement time was set at 30 s and five independent records were collected for each sample. Results were analyzed using NTA 3.1. software and calculated according to the dilutions used. The mean results ± SD were presented on graphs.

Additionally, Western blot (WB) analysis of EV markers was performed. For this purpose, for each cell line, whole-cell protein extracts (prepared as described in [19]) and ectosome samples (containing 50 μg of proteins according to MicroBCA method) were separated on 10% SDS-PAGE stain-free precast gels in reducing conditions and transferred to the PVDF membrane. The purity of ectosome samples was assessed with the use of anti-CD63 (1:2000), anti-HSP70 (1:2000) and anti-ARF6 (1:500) antibodies. After 1 h incubation with primary antibodies, anti-mouse IgG-HRP (1:400) was used as a secondary antibody. Markers were detected using chemiluminescent substrates for HRP and ChemiDoc Imaging System (Bio-Rad).

### 4.4. LC–MS/MS Proteomics

Ectosome lysis, sample preparation for mass spectrometric analysis, nanoLC–MS/MS, and analysis of proteomic data were performed as described below and in [19].

#### 4.4.1. Ectosome Lysis

The ectosome pellets were washed three times with PBS and suspended in 50 µL of lysis buffer (100 mM Tris-HCl pH 7.6, 1% SDS). Lysates were sonicated with Bioruptor UCD-200 (Diagenode, Seraing, Belgium) for 20 min at high intensity (320 W, 30 s/30 s on/off). Next, the samples were denatured at 95 °C under strong agitation for 5 min and centrifuged at 20,000× *g* for 10 min at 20 °C. Proteins were precipitated by adding one volume of trichloroacetic acid (TCA) to four volumes of the sample. After overnight incubation at –20 °C, the samples were spun at 10,000× *g* for 15 min at 10 °C and washed two times with ice-cold acetone. The pellets were resuspended in 100 µL of 10 mM HEPES pH 8.5.

#### 4.4.2. Sample Preparation for Mass Spectrometric Analysis

The samples were prepared using paramagnetic bead technology based on the Single-Pot Solid-Phase-Enhanced Sample Preparation (SP3) [39]. GE45152105050250 and GE65152105050250 SpeedBeads™ mixed at a 1:1 ratio were used. The proteins were reduced with dithiothreitol, alkylated with iodoacetamide, and digested with Trypsin/Lys-C Mix.

#### 4.4.3. Liquid Chromatography and Tandem Mass Spectrometry (LC-MS/MS)

Peptides were analyzed using an UltiMate 3000 RSLCnano System coupled with a Q-Exactive mass spectrometer (Thermo Fisher Scientific) with DPV-550 Digital PicoView nanospray source (New Objective). The sample was loaded onto a trap column (Acclaim PepMap 100 C18, 75 μm × 20 mm, 3 μm particle, 100 Å pore size) in 2% acetonitrile with 0.05% trifluoroacetic acid (TFA) at a flow rate of 5 μL/min, and further resolved on an analytical column (Acclaim PepMap RSLC C18, 75 µm × 500 mm, 2 µm particle, 100 Å pore size) with a 90 min gradient from 2% to 40% acetonitrile in 0.05% formic acid at a flow rate of 200 nL/min. The Q-Exactive was operated in a data-dependent mode using the top eight method. Full-scan MS spectra were acquired at a resolution of 70,000 at *m*/*z* 200 with automatic gain control (AGC target) of 1 × 10^−6^. The MS/MS spectra were acquired at a resolution of 35,000 at *m*/*z* 200 with an AGC target of 3 × 10^−6^. The maximum ion accumulation times for the full MS and the MS/MS scans were 120 ms and 110 ms, respectively. Peptides were dynamically excluded from fragmentation within 30 s. Three technical replicates for each of the two biological samples were measured.

#### 4.4.4. Analysis of Proteomic Data

The RAW files were processed by the Proteome Discoverer platform (v.1.4, Thermo Fisher Scientific) and searched against the SwissProt database with *Homo sapiens* taxonomy restriction (release February 2020, 20366 sequences) using a locally installed MASCOT search engine (v. 2.5.1, Matrix Science). The following parameters were applied: fixed modification, cysteine carbamidomethylation; variable modifications, methionine oxidation and protein *N*-terminal acetylation; peptide mass tolerance, 10 ppm; fragment mass tolerance, 20 mmu. Only tryptic peptides with up to one missed cleavage were considered. Target Decoy PSM Validator was applied with the maximum false discovery rate (FDR) for peptides set to 0.01. The mass spectrometry data were deposited to the ProteomeXchange Consortium [40] via the MassIVE repository with the dataset identifier PXD025825.

### 4.5. Bioinformatic Analysis

Proteins identified by both biological repetitions of ectosome samples (that is, in every technical repetition) and with at least two peptides were chosen manually to create the final protein lists. Venn diagrams, including Vesiclepedia protein overlap, and gene ontology (GO) analysis, with regard to the cellular compartment, molecular function and biological processes were performed with the use of FunRich 2.0 software with protein UniProt (release 2020_12) database as a reference. For each GO term, ten categories with the highest statistical significance of protein enrichment within the respective category (calculated as −log10(*p*-value)) were presented on graphs. Entire GO data is provided in Supplementary Data S2.

Relative quantification of proteins was determined based on normalized spectrum abundance factors (*d*NSAF; [24]), weighting distribution of unique and shared peptide-spectrum matches (PSMs). The equation used to calculate *d*NSAF was as follows:

$$d\mathrm{NSAF}=\frac{\mathrm{uSpc}+\left[\left(d\right)\left(\mathrm{sSpC}\right)\right]}{\mathrm{uL}+\mathrm{sL}}\;d=\frac{\mathrm{uSpC}}{\sum \mathrm{uSpC}}$$

where spectral counts from peptides uniquely mapping to a protein are denoted as “uSpC”, and spectral counts from peptides shared between isoforms are labeled “sSpC”. Protein amino acid lengths mapping to unique and shared peptides are denoted as “uL” and “sL”; “*d*” denotes distribution factor.

Interactomes were prepared using string v11.0 (https://string-db.org, accessed on 23 April 2021).

### 4.6. Wound Healing Assay

HCV-29 and T-24 cells were cultured to confluence on 6-well plates. Subsequently, the cell-coated surface was scraped with a 200 µL pipette tip and two different doses (30 µg and 60 µg of proteins) of ectosomes were added for 18 h of incubation. Each wound was photographed in 10 separate fields immediately after scraping (0 h) and after 18 h. The average rate of wound closure was evaluated by multiple measurements of the wound width using Zeiss AxioVision Rel.4.8 image analysis software and calculated as follows:

wound closure = (initial wound width (0 h) − wound width after 18 h)/initial wound width (0 h)

Results were standardized in relation to the untreated control (taken as 1).

### 4.7. Alamar Blue Cell Viability Assay

HCV-29 and T-24 cells were seeded onto 96-well plates at the density of 1 × 10^4^ cells/100 µL. The next day, a serum-free medium was added and cells were incubated with two doses of ectosomes (30 µg and 60 µg of proteins). After 18 h of incubation, 10% of Alamar Blue reagent was added to each well and after 2 h fluorescence intensity was measured at 560/595 nm. Results were standardized in relation to the untreated control (taken as 1).

### 4.8. Statistical Analysis

Three repetitions of Alamar Blue and wound healing assays were performed for each experimental setting. Analysis of variance (one-way ANOVA) and post-hoc Tukey’s test were later performed with the use of Statistica 12 software to test for statistically significant differences with *p*-value < 0.05.

## Figures and Tables

**Table 1:** Functional classification of cancer-related proteins identified in ectosomes derived from T-24 urothelial bladder carcinoma cells.

Cell Proliferation (GO:0008283)	Cell Adhesion (GO:0007155)	Cell Migration (GO:0016477)	Angiogenesis (GO:0001525)	Immune Response (GO:0006955)	Drug Response (GO:0042493)
Double-strand break repair protein MRE11 (MRE11) Annexin A7 (ANXA7) Guanine nucleotide-binding protein G(i) subunit alpha-2 (GNAI2) Ras-related C3 botulinum toxin substrate 1 (RAC1) DAZ-associated protein 1 (DAZAP1) Myosin-10 (MYH10) Guanine nucleotide-binding protein G(I)/G(S)/G(T) subunit beta-1 (GNB1) Interleukin-18 (IL18) Thioredoxin reductase 1, cytoplasmic (TXNRD1) X-ray repair cross-complementing protein 5 (XRCC5)	ADP-ribosylation factor 6 (ARF6) Amyloid-beta precursor protein (APP) Catenin alpha-1 (CTNNA1) Catenin beta-1 (CTNNB1) CCN family member 1 (CCN1) CD166 antigen (ALCAM) CD44 antigen (CD44) Cell surface glycoprotein MUC18 (MCAM) Collagen alpha-1(XVIII) chain (COL18A1) EGF-like repeat and discoidin I-like domain-containing protein 3 (EDIL3) Ephrin type-A receptor 2 (EPHA2) Flotillin-2 (FLOT2) Intercellular adhesion molecule 1 (ICAM1) Neural cell adhesion molecule L1 (L1CAM) Ras-related C3 botulinum toxin substrate 1 (RAC1) Sarcoplasmic/endoplasmic reticulum calcium ATPase 2 (ATP2A2) Sodium/potassium-transporting ATPase subunit beta-1 (ATP1B1) Sphingosine 1-phosphate receptor 1 (S1PR1) Transforming growth factor beta-1-induced transcript 1 protein (TGFB1I1) Transmembrane 9 superfamily member 4 (TM9SF4)	Asparagine--tRNA ligase, cytoplasmic (NARS1) CD44 antigen (CD44) Coronin-1B (CORO1B) Coronin-1C (CORO1C) Ephrin type-A receptor 2 (EPHA2) Fascin (FSCN1) Microtubule-associated protein RP/EB family member 1 (MAPRE1) Neural cell adhesion molecule L1 (L1CAM) Pre-mRNA-processing factor 40 homolog A (PRPF40A) Rho-related GTP-binding protein RhoC (RHOC) Sphingosine 1-phosphate receptor 1 (S1PR1) Transforming protein RhoA (RHOA)	Caveolin-1 (CAV1) Cell surface glycoprotein MUC18 (MCAM) Collagen alpha-1(XVIII) chain (COL18A1) Collagen alpha-2(IV) chain (COL4A2) E3 ubiquitin-protein ligase RNF213 (RNF213) Endoplasmic reticulum aminopeptidase 1 (ERAP1) Interleukin-18 (IL18) Programmed cell death protein 6 (PDCD6) Sphingosine 1-phosphate receptor 1 (S1PR1) Tryptophan-tRNA ligase, cytoplasmic (WARS1)	Complement component 1 Q subcomponent-binding protein, mitochondrial (C1QBP) Deoxynucleoside triphosphate triphosphohydrolase SAMHD1 (SAMHD1) HLA class I histocompatibility antigen, A alpha chain (HLA-A) Interferon-induced transmembrane protein 3 (IFITM3) Purine nucleoside phosphorylase (PNP)	116 kDa U5 small nuclear ribonucleoprotein component (EFTUD2) CAD protein (CAD) Nucleoside diphosphate kinase A (NME1) Transferrin receptor protein 1 (TFRC)

## Data Availability

MS/MS data are available via ProteomeXchange with identifier PXD025825.

## Supplementary Materials

Supplementary materials can be found at https://www.mdpi.com/article/10.3390/ijms22136816/s1.

## Abbreviations

ECM	Extracellular Matrix
EDIL 3	EGF-Like Repeat and Discoidin I-like Domain-Containing Protein 3
EMT	Epithelial-Mesenchymal Transition
EVs	Extracellular Vesicles
FASN	Fatty Acid Synthase
GO	Gene Ontology
LC-MS/MS	Liquid Chromatography Coupled to Tandem Mass Spectrometry
NTA	Nanoparticle Tracking Analysis
TEM	Transmission Electron Microscopy
UBC	Urothelial Bladder Carcinoma
